# Hemoparasitism in grazing cattle and risk factors associated with husbandry management in an endemic area of Eastern Colombia

**DOI:** 10.1007/s12639-024-01723-w

**Published:** 2024-08-19

**Authors:** Natalie Hell Mor, Julieth Viviana Montenegro Tavera, Julio César Tobón, Blanca Lisseth Guzmán Barragán, Giovanny Beltran López, Jimmy Jolman Vargas Duarte, Danny Wilson Sanjuanelo Corredor, Gabriel Andrés Tafur-Gómez

**Affiliations:** 1 Laboratorio de Parasitologia, Universidad de Ciencias Aplicadas y Ambientales – U.D.C.A, Calle 222 # 55-37, Bogotá D.C 111166,, Bogotá, Colombia; 2 Empresa Colombiana de Productos Veterinarios – VECOL, Bogotá, Colombia; 3 Universidad Nacional de Colombia, Instituto de Genética, Bogotá, Colombia

**Keywords:** Vector-borne diseases, *Anaplasma*, *Babesia*, *Trypanosoma*, Co-infection, Susceptibility

## Abstract

Vector-borne pathogens induce hemoparasitism in cattle causing substantial economic losses in tropical and subtropical areas. Infectious cattle actively contribute to maintaining the transmission cycle, and the presence of these animals must be associated with husbandry management and environmental changes. In the present study, we conducted a cross-sectional study sampling 1,000 bovines to identify infectious cattle diagnosed by a direct technique and employed a dichotomic questionnaire for association analyses, hierarchical clustering, and Principal Component Analysis (PCA). Overall prevalence with infectious cattle was 34.99%, where 97% of the farms had at least one infectious animal per genera, and the prevalence in properties ranged between 16.39 and 53.85%. Of these animals, 26.20% tested positive for *Anaplasma sp.*, 8.40% for *Babesia* spp., and 1.30% for *Trypanosome* spp. The main co-infection showed 5% *Anaplasma* sp. *– Trypanosome* spp., followed by 4% *Babesia* spp. – *Trypanosome* spp. These bovines showed association with the use of the Jersey breed (OR = 2.016 C.I:1.188–3.419), selling animals for replacement (OR = 1.417 CI:1.022–1.965), participation in livestock exhibitions (OR = 2.009 CI:1.262–3.199), premises with burials (OR = 2,064 CR: 1.414–3.011), use of palm kernel (OR = 1.935 C.I:1.198–3.124), and the use of ivermectin (OR = 1.548 CI: 1.085–2.210) as a susceptibility. The hierarchical clustering revealed clusters among properties with different hemoparasite prevalence, with notable co-infections observed. The subsequent PCA identified that significant risk factors contributed to hemoparasitism positivity. We conclude that infectious cattle in the endemic area showed an association with husbandry management that permits the success of vector and maintenance of the enzootic or epizootic cycle in the herds.

## Introduction

Vector-borne pathogens in cattle lead to substantial economic losses globally, with a higher prevalence of protozoan and rickettsial agents in tropical and subtropical regions (Uilenberg 1995). The primary etiologies that affect cattle in these regions belong to the *Babesia*, *Anaplasma*, and *Trypanosoma* genera. The transmission of these occurs by ixodid ticks and biting flies, with similar distribution patterns throughout many parts of the world (Vokaty et al. 1996). In South American countries, co-infestations of ectoparasites are linked to co-infections of hemoparasites in cattle (Paoletta et al. 2018). Therefore, the spread of these agents is influenced by epidemiological factors that may involve the production system and management, leading to reduced productivity, increased veterinary costs, and economic losses (Behar et al. 2020). The presence of infectious cattle in herds, such as persistently infected animals, depends on the degree of endemism in a population that facilitates the transmission of agents in the co-circulation of vectors (Mahoney and Ross 1972). However, in South American countries, varying levels of prevalence, including stable or unstable enzootic endemism, predispose to enzootic cycle or epizootic outbreaks in the presence of infectious cattle (Ferreira et al. 2022).

In Colombia, the premises show a notable difference in rainfall and climate conditions with temperature and humidity according to the altitudes in the mountain range and the extensive and semi-extensive management and the variation in climate conditions favour the development of hematophagous arthropods in cattle herds (Cerón et al. 2022). The ectotherm arthropods include *Rhipicephalus (Boophilus) microplus*, *Tabanus nebulosus*,* T. pungens*, *T. claripennis*,* T. occidentalis*,* Stomoxys calcitrans*, and *Haematobia irritans* are the main ectoparasites that affect the cattle. These arthropods co-circulate in the regions with the highest incidences of 0 to 2,600 msnm (Otte and Abuabara 1991; Otte et al. 1994; Márquez 2003; Parra-Henao et al. 2008; Vecino et al. 2010). The herds located in areas with co-infestation of those ectoparasites show variable enzootic endemism involving agents such as *Babesia bovis*, *Babesia bigemina*,* Anaplasma marginale*, *Trypanosoma vivax* y *Trypanosoma evansi* in cattle in single or co-infections (Wells et al. 1970; Corrier et al. 1978; Patarroyo et al. 1978). However, the epidemiological situations of these pathogens were traditionally associated with ecological conditions with a scant relation to management husbandry in cattle herds.

Similarly, these epidemiologic situations have historical diagnoses by serologic methods that showed accurate results to previously exposed animals (Nayel et al. 2012). These focused on single-agent infections with little attention to co-infections in cattle. In the endemic areas, PCR techniques confirmed the agents previously identified by light microscopy, showing fewer positive animals than those obtained by serological methods, providing a similar diagnostic value to microscopy (Valkiunas et al. 2008; Nayel et al. 2012; Jaimes-Dueñez et al. 2017). However, despite advancements in serologic and PCR techniques there persists a difficulty in detecting infectious animals, leading to their underrepresentation in population surveys (Nayel et al. 2012). As a result, traditional parasitological methods like blood smears remain required for identifying blood parasites and permit the diagnosis of infectious animals with low parasitemic charge (Böse et al. 1995; Moody and Chiodini 2000). Likewise, blood smears are among the most accessible and cost-effective methods for the diagnosis and timely detection of hemoparasitic infections (Böse et al. 1995).

The diagnosis of infectious animals and their association with risk factors may aid in designing control strategies that involve active animals participating in the transmission cycle (Antonio Alvarez et al. 2019). Detecting hemoparasite co-infection in infectious cattle facilitates better comprehension of parasite associations in pathogen transmission and reproduction in endemic areas (Vaumourin et al. 2015). Less sensitive diagnostic methods, like blood smear, can assist in identifying highly parasitemic cattle, including those that are repeatedly and persistently infected. These animals actively contribute to the transmission cycle by maintaining parasitism within the herd (Mahoney and Ross 1972; Antonio Alvarez et al. 2019). However, the evaluation of hemoparasites in single or co-infections among infectious cattle related to husbandry management is still insufficient in many parts of the country. This study aimed to determine the prevalence of infectious cattle infected with hemoparasites in either a single or co-infection associated with risk factors in an endemic region of eastern Colombia. The findings may assist in enhancing prevention plans and intervention strategies for livestock affected by vector-borne pathogens.

## Materials and methods

### Study area

The study was carried out in the rural area of Villavicencio district, geographically set at N 4°08′33″ O 73°37′46″ in the Meta department in the eastern region of Colombia. This region is located at an altitude of 380 m.s.n.m. and has an average temperature of 27 °C. The rain patterns are mixed, with two wet seasons varying from March to June and September to October. This pattern has a yearly rainfall between 3,000 and 4,000 mm. Dry seasons in this region are likely to occur from November to Febuary and from July to August (Urrea et al. 2019). The highest precipitation levels occur in the first season and moderate in the year’s second season, reaching 80% relative humidity (IDEAM 2014; Urrea et al. 2019). This region is characterized by diverse topography, featuring vast plains, lowlands, and some high hilly areas. It is part of the Orinoco River basin, contributing to its predominantly flat landscape with gentle undulations (IDEAM 2014; Pacheco et al., 2019).

### Collection and processing of samples

A cross-sectional epidemiological survey was designed involving grazing cattle under extensive and semi-extensive husbandry, having several breeds, sex, different ages, and sharing with other animal species the same farms and area. We calculated the sample size including the bovine population census of Villavicencio, estimated at 108,109 cattle by the ICA (2016), and a seroprevalence of 66.1% for hemoparasites identified by Mateus and Vizcaíno (1986) within the same district. We considered a confidence interval of 95% and a design effect of 3% according to Dohoo (2003). This data was processed in Epi Info™ v 7.2.0.1 software (CDC, 2016) estimating a sample size of 1023 animals. However, to account for potential field losses, we randomly sampled 1,000 bovines in 5 villages and 29 farms between September and December 2017. In this period of sampling, the animals were exposed to weather conditions of the second humid period, with highest infestation of biting flies during rainfall, and highest infestation of ixodid ticks in the transitional period (Betancourt 1990; Cassalett et al. 2013). Considered the vector borne transmission and the presence of infectious cattle, blood was collected from the tail vein in EDTA tubes and preserved at 4 °C. Posteriorly, we mounted the blood smears, dried, and fixed with methanol, and stained them with Wright adding some drops of distilled water for 4 min. Then, the slides were washed with water and left to dry vertically to analyze in a 100X objective with immersion oil according to the method described by Marshall et al. (1975).

### Data analysis

The prevalence was calculated considering a confidence interval (CI) of 95% with data input via the https://sites.google.com/site/seriescol/shapes platform and analyzed using ESRI ArcGIS^®^ v10.1 geographic information system (GIS) software for the construction of epidemiological maps. Data collected from an epidemiological questionnaire, featuring dichotomous responses, were utilized for association analysis. Univariate analysis, assessing the relationship between *Anaplasma* spp., *Babesia* spp., and *Trypanosoma* spp. positivity, was conducted using Pearson’s Chi-square test. Subsequently, a multivariable logistic regression model was employed, incorporating variables with a significance level of *p* ≤ 0.05, based solely on the univariate analysis of the overall diagnosis for hemoparasites (at least one hemoparasite per animal). Statistical analyses were performed using SPSS version 20 software (SPSS Inc., Chicago, II, USA).

For cluster analysis we considered the prevalence of each parasite condition, positive proprieties, and risk factors identified. The dendrogram plot R package was utilized for analysis, incorporating property dates and prevalence results. Similarly, the Scatted Plot R package was used, inserting propriety data and positivity to *Anaplasma* spp., *Babesia* spp. and *Trypanosoma* spp. Additionally, principal component analysis (PCA) was conducted employing the Biplot R package, accentuating the significance of risk factors identified as variables. Finally, clustering was contrasted for each propriety concerning the prevalence of each hemoparasite.

## Results

### Prevalence of hemoparasites

From 1,000 cattle sampled, 334 tested positive for almost one hemoparasite genus showing a total prevalence of 33.40% (95% CR:29.96–37.13). For each genus, these animals showed 26.20% (95% CR:23.17–29.52) for *Anaplasma* spp., 8.40% (95% CR:6.742–10.35) for *Babesia* spp., and 1.30% (95% CR:0.723–2.167) for *Trypanosome* spp. Out of 1,000 animals tested, 27 showed co-infection, resulting in a prevalence rate of 2.7% for *Anaplasma* spp. *– Trypanosome* spp., 4% (4/1,000) *Babesia* spp. – *Trypanosome* spp., 1.7% (17/1000) for *Anaplasma* spp. *– Babesia* spp., and only one animal 1% (1/1,000) for three genera combined. According to sex and age from positive cattle, the effect of sex showed a representative prevalence by almost one hemoparasite and *Trypanosoma* spp. About each hemoparasite positivity, the cattle aged until one year old and older than three years showed the most representative prevalence by *Anaplasma* spp. For *Babesia* spp., the cattle older than three years old showed the most representative prevalence, and for *Trypanosome* spp., the cattle until one year old showed the most representative prevalence (Table 1).

**Table 1:** Prevalence of vector-borne pathogens in cattle by sex and age in Villavicencio - Colombia

Agent	Sampled animals (*n*)	Positive animals	Prevalence %	C.I
*Hemoparasites*	1000	334	33.40	29.96–37.13
Females	803	281	34.99	31.08–39.27
Males	197	53	26.90	20.36–34.92
< 1 year old	226	75	33.19	26.29–41.36
1–2-year-old	106	33	31.13	21.79 43.21
2–3-year-olds	81	17	20.99	12.63–32.92
> 3-year-old	587	209	35.60	31.02–40.69
*Anaplasma sp*	1000	262	26.20	23.17–29.52
Females	803	221	27.52	24.07–31.34
Males	197	41	20.81	15.13–27.96
< 1 year old	226	64	28.32	21.99–35.93
1–2-year-old	106	26	24.53	16.36–35.43
2–3-year-olds	81	14	17.28	09.83–28.31
> 3-year-old	587	158	26.92	22.96–31.37
*Babesia spp*	1000	84	8.40	6.742–10.35
Females	803	68	8.47	6.627–10.67
Males	197	16	8.12	4.808–12.91
< 1 year old	226	15	6.64	3.85–10.70
1–2-year-old	106	7	6.60	2.88–13.06
2–3-year-olds	81	4	4.94	1.56–11.91
> 3-year-old	587	58	9.88	7.57–12.68
*Trypanosoma spp*	1000	13	1.30	0.723–2.167
Females	803	9	1.12	0.546–2.057
Males	197	4	2.03	0.645–4.898
< 1 year old	226	5	2.21	0.81–4.90
1–2-year-old	106	-	0.00	-
2–3-year-olds	81	-	0.00	-
> 3-year-old	587	8	1.36	0.63–2.58

### Hemoparasites in cattle by villages and properties

**Fig. 1:**
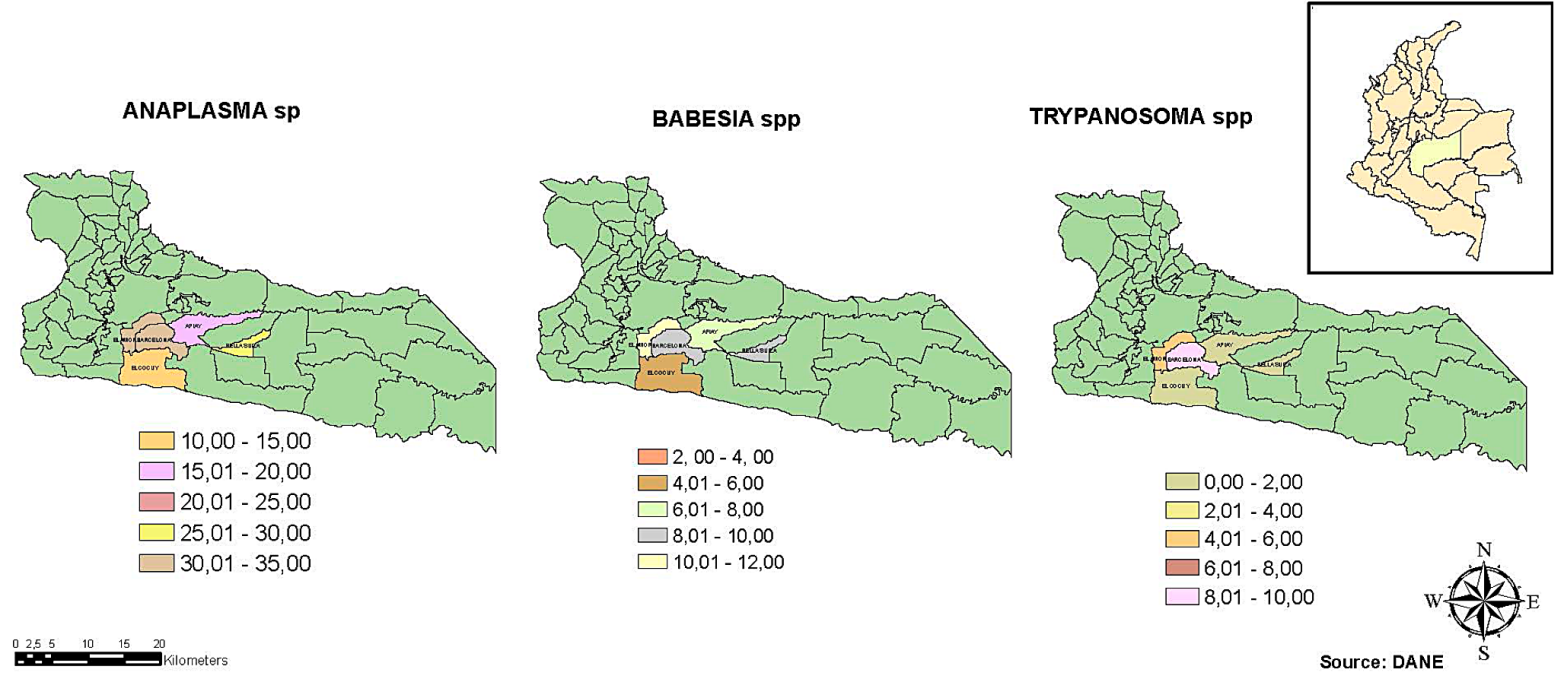
Prevalence rate and affectation area by cattle vector-borne pathogens in Villavicencio-Colombia

In the village Amor, the prevalence of hemoparasites showed a rate of 38.58% (95% CR:30.61–48.02), in Apiay 25.44% (95% CR:18.65–33.96), in Barcelona 38.01% (95% CR:30.51–46.82), in Bella Suiza 35.65% (95% CR:29.51–42.69) and Cocuy 18.75% (95%CR:11.46–29.06). The villages with a higher prevalence of *Anaplasma* spp. were Amor with 31.98% (95% CR:24.78–40.65) and Barcelona with 31.22% (95% CR:24.48–39.28), *Babesia* spp., Amor 10.15% (95% CR:06.37–15.40) and for *Trypanosoma* spp., the village of Barcelona with 9.05% (95% CR:1.517–29.9). Similarly, the prevalence was calculated in each property, identifying that 97% of the farms had at least one positive animal per parasite genera and both enzootic endemism stable (< 30%) and unstable (> 30%) considering the prevalence (Table 2). The overall hemoparasites in the properties ranged between 16.39 and 53.85%, the prevalence of *Anaplasma* spp. ranged between 8.20 and 54.00%, the prevalence of *Babesia* spp. was between 5.00 and 25.00%, and the prevalence of *Trypanosome* spp. ranged from 0.00 to 15.38% (see Fig. 1).

**Table 2:** Prevalence of infectious animals by each property including each condition of hemoparasitism

Properties	Sampled cattle	Overall prevalence	Prevalence of Anaplasma sp.	Prevalence of Babesia spp.	Prevalence of Trypanosoma spp.
Barcelona	51	45.10	33.33	15.69	1.96
Bariri	14	28.57	28.57	7.14	0.00
Base aerea	24	45.83	25.00	20.83	0.00
Bella vista	40	52.50	45.00	10.00	0.00
Brisas del ocoa	24	37.50	37.50	8.33	0.00
Campo hermoso	50	20.00	12.00	8.00	2.00
El palmar	19	42.11	31.58	15.79	0.00
El recreo	4	25.00	0.00	25.00	0.00
El refugio	53	32.08	26.42	7.55	0.00
El rocio	5	40.00	40.00	0.00	0.00
El rodeo	18	44.44	38.89	5.56	0.00
El hachon	80	26.25	23.75	6.25	1.25
La feria	28	25.00	25.00	0.00	0.00
La fortuna	49	46.94	42.86	4.08	0.00
La fortuna iv	33	42.42	33.33	9.09	0.00
La palma	61	16.39	8.20	6.56	3.28
La primavera	25	48.00	44.00	8.00	4.00
Las garzas	10	30.00	20.00	10.00	0.00
Las margaritas	36	30.56	22.22	11.11	0.00
Marsella	13	53.85	15.38	23.08	15.38
Mi llanura	46	17.39	17.39	0.00	0.00
Nieves	30	33.33	16.67	20.00	3.33
San francisco	10	20.00	10.00	10.00	0.00
San marcos	46	43.48	28.26	15.22	4.35
San pedro	40	47.50	37.50	12.50	0.00
Santa ana	65	26.15	23.08	3.08	1.54
Santa lucía	10	0.00	0.00	0.00	0.00
Universidad de los llanos	16	18.75	18.75	6.25	0.00
Urania	100	33.00	27.00	5.00	1.00

### Risk factors

Considering the positivity for at least one hemoparasite by univariate analysis, the relevant risk factors showed the use of Jersey breed (OR = 2.016 C.I:1.188–3.419), the participation in livestock exhibitions (OR = 2.009 CI:1.262–3.199), farms with burials (OR = 2,064 CR: 1.414–3.011), use of palm kernel (OR = 1.935 C.I:1.198–3.124), and the use of ivermectin (OR = 1.548 CI: 1.085–2.210) (Table 3). On the other hand, the multivariate association analysis revealed, as risk factors, farms that had burials (OR = 1.757 C. I: 1.088–2.838) and those that participated in livestock exposition (OR = 2,043 C.I: 1.088–2.838). However, the same analysis showed as a protective factor the use of the best management breed, which is Angus (OR = 0,504 C.I: 0.310–0.820), and gastrointestinal deworming with benzimidazole (OR = 0,611 C.I: 0.422–0.885). However, the same analysis showed as protective factors the use of the best management breed Angus (OR = 0,504 C.I: 0.310–0.820) and gastrointestinal deworming with benzimidazole (OR = 0,611 C.I: 0.422–0.885). Considering the positivity of each genera parasite processing data by univariate analysis, the unique risk factors associated with *Anaplasma* spp. showed the presence of canines in the farms (OR = 1,663 C.I: 1.162–2.379). The particular risk factors associated with *Babesia* spp. showed the presence of bovines older than 3 years old (OR = 1.632 C.I: 1.009–2.639), the use of crossbreed cattle (OR = 1.619 C.I: 1.008–2.602), females inseminated outside the farms (OR = 1.856 C.I: 1.166–2.954) and the purchase of animals for replacement (OR = 1,930 C.I: 1.043–3.571). The unique risk factors involving *Trypanosome* spp. showed the cattle sharing grazing areas with buffaloes (OR = 16.13 C. I: 3.194–81.485) (Table 3).

**Table 3:** Univariate analysis of risk factors associated with vector-borne pathogens in cattle from Villavicencio -Colombia

Agent	General Variables	X2	*P*	OR	C.I
*Hemoparasites*	Females	4.655	0.031*	1.463	1.034–2.069
	Males	4.655	0.031*	0.684	0.483–0.967
	Jersey	6.994	0.008	2.016	1.188–3.419
	Angus	7.817	0.005	0.554	0.364–0.842
	Entry of different animal species to the premises	4.937	0.026	0.690	0.496–0.958
	Participate in livestock exhibition	8.924	0.003	2.009	1.262–3.199
	Burials	14.533	< 0.001	2.064	1.414–3.011
	Palm kernel supplement	7.490	0.006	1.935	1.198–3.124
	Use of ivermectin	5.852	0.016	1.548	1.085–2.210
	Use of benzimidazol	6.492	0.001	0.710	0.545–0.924
*Anaplasma* sp	Females	3.683	0.055	1.445	0.991–2.107
	Males	3.683	0.055	0.692	0.475–1.009
	Jersey	6.797	0.009	2.025	1.181–3.475
	Canines on the premises	7.853	0.005	1.663	1.162–2.379
	Entry of different animal species to the premises	8.286	0.004	0.583	0.403–0.844
	Participate in livestock exhibition	4.115	0.043	1.646	1.013–2.676
	Palm kernel alimentation	7.451	0.006	1.963	1.201–3.210
	Use of ivermectin	9.206	0.002	1.860	1.240–2.790
	use of benzimidazol	5.296	0.021	0.717	0.540–0.952
*Babesia* spp	> 3 years old	4.05	0.044	1.632	1.009–2.639
	Crossbreed breed	4.029	0.045	1.619	1.008–2.602
	Angus	10.167	0.001	0.139	0.034–0.571
	Cows inseminated outside the farms	6.972	0.008	1.856	1.166–2.954
	Buffaloes on the premises	3.687	0.055	3.356	0.905–12.437
	Buys beef cattle for fattening	3.607	0.058	1.567	0.983–2.498
	Buys animals for replacement	4.529	0.033	1.930	1.043–3.571
*Trypanosoma* spp	Buffaloes on the premises	20.364	< 0.001	16.13	3.194–81.485
	Canines on the premises	10.259	0.001	0.191	0.062–0.589
	Participate in livestock exhibition	4.274	0.039	3.648	0.983–13.541

*Note* OR: Odds Ratio; CR: Confidence rate (95%), *Statistically significant

### Cluster analysis

**Fig. 2:**
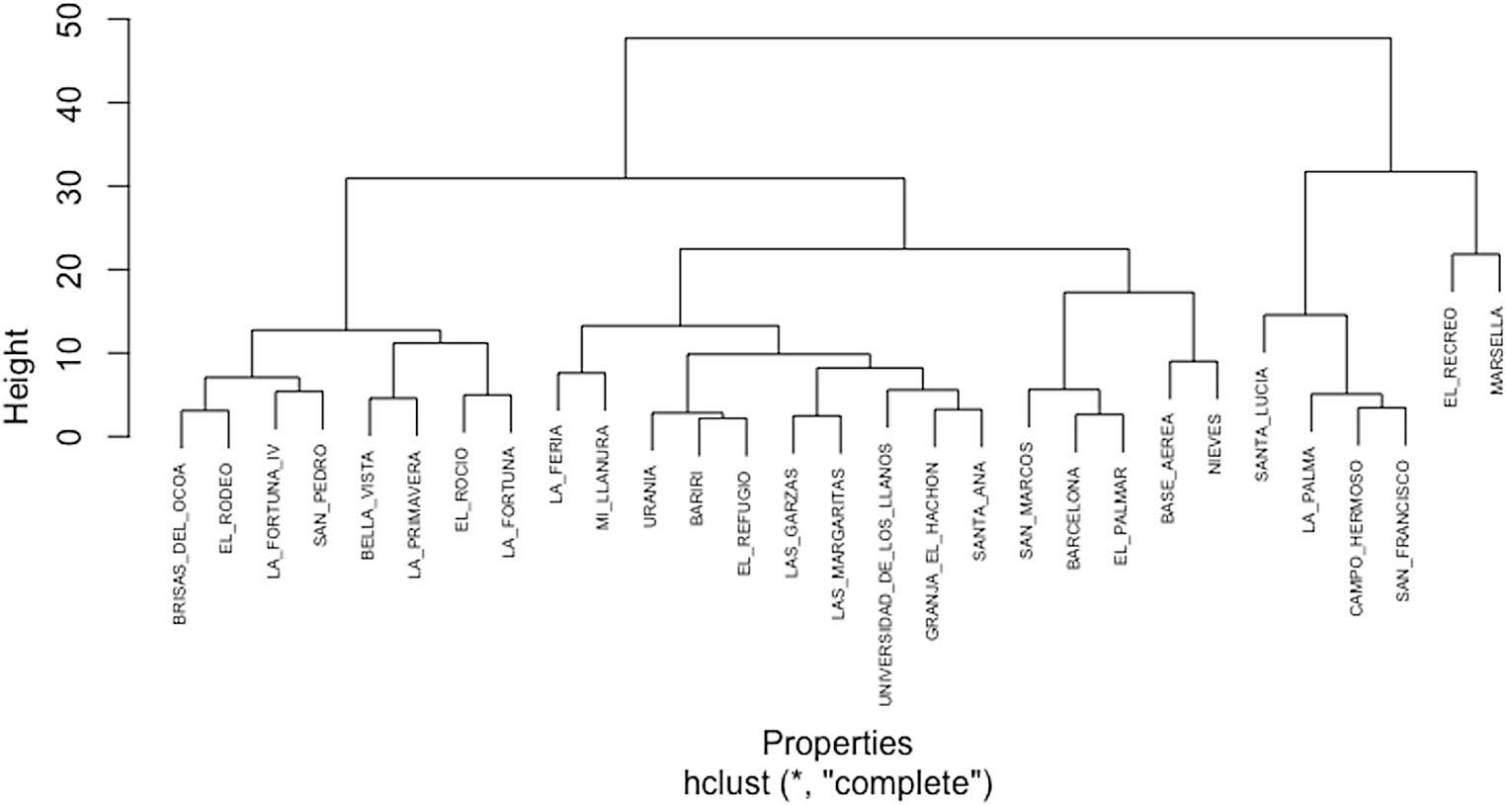
Dendogram of properties grouped by the prevalence of hemoparasites: This plot illustrates a multivariate analysis conducted on properties identified as positive for hemoparasites. The properties under consideration are Barcelona, Bariri, Base Aerea, Bella Vista, Brisas del Cocoa, Campo Hermoso, El Palmar, El Recreo, El Refugio, El Rocio, El Rodeo, Granja El Hachon, La Feria, La Fortuna, La Fortuna IV, La Palma, La Primavera, Las Garzas, Las Margaritas, Marsella, Mi Llanura, Nieves, San Francisco, San Marcos, San Pedro, Santa Ana, Santa Lucía, Universidad de los Llanos y Urania. The longer the distance the fewer similarities between the properties, the closer they are more similarities the properties must present hemoparasites

**Fig. 3:**
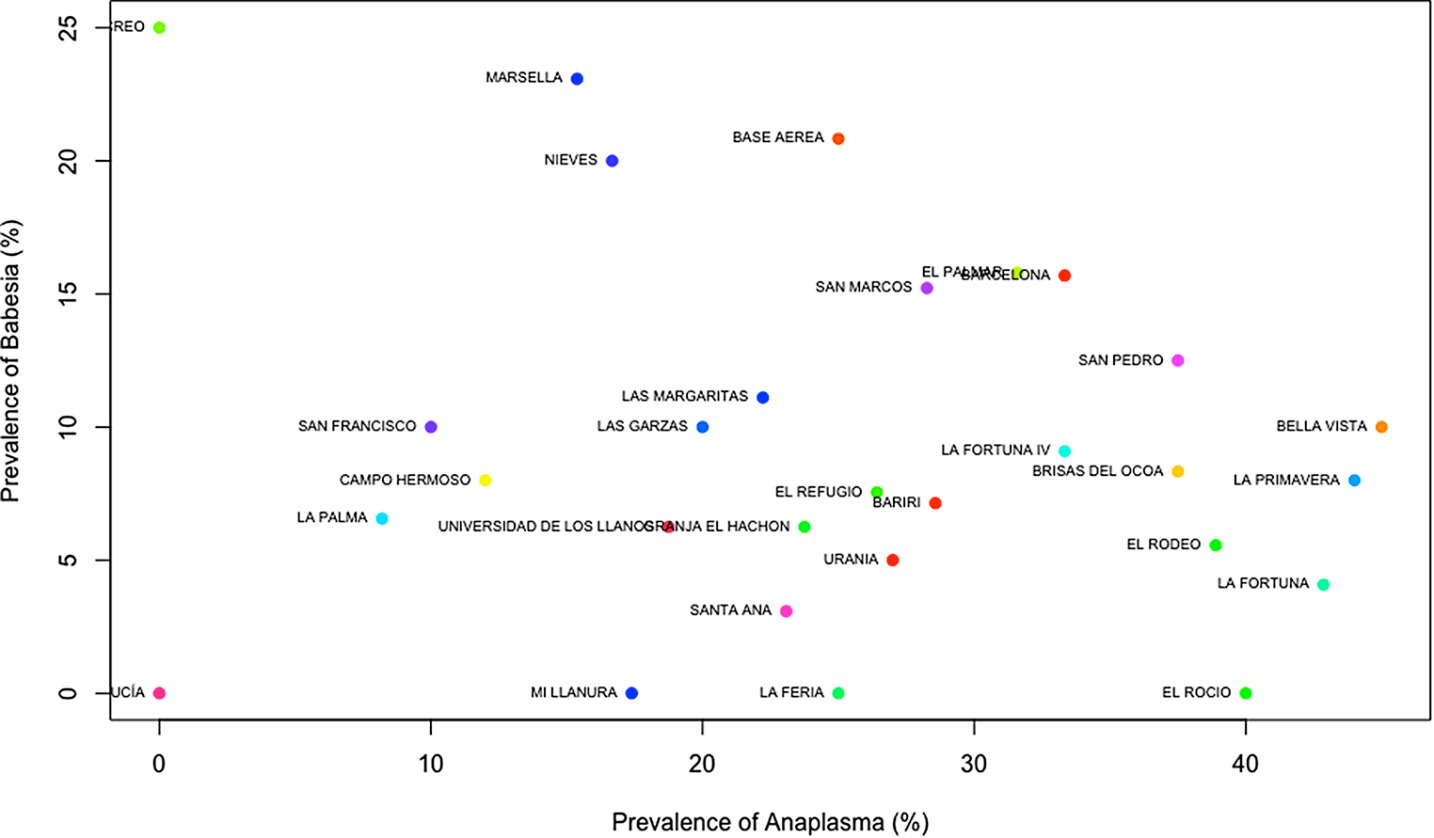
Scatter Plot of properties by the prevalence of *Anaplasma* and *Babesia*: Each point on the scatter plot corresponds to a specific property, with the X and Y axes representing dimensions derived from factor analysis (linear combinations of variables). The proximity between points signifies similarity in the prevalence of *Anaplasma* sp. and *Babesia* spp. The observed clusters indicate properties that share similar characteristics. The properties included in this analysis are: Barcelona, Bariri, Base Aerea, Bella Vista, Brisas de la cocoa, Campo Hermoso, El Palmar, El Recreo, El Refugio, El Rocio, El Rodeo, Granja El Hachon, La Feria, La Fortuna, La Fortuna IV, La Palma, La Primavera, Las Garzas, Las Margaritas, Marsella, Mi Llanura, Nieves, San Francisco, San Marcos, San Pedro, Santa Ana, Santa Lucía, Universidad de los Llanos and Urania

**Fig. 4:**
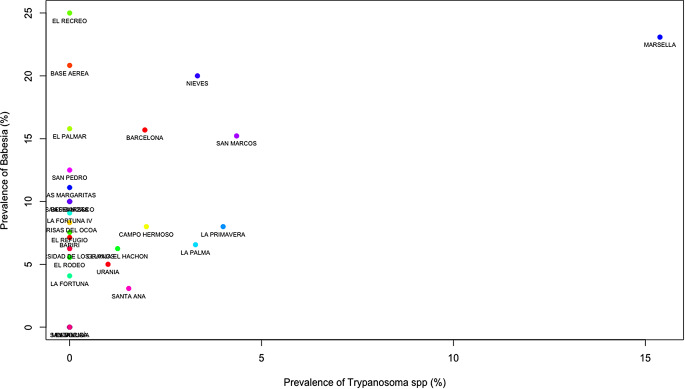
Scatter Plot of properties by the prevalence of *Trypanosoma* and *Babesia*: The points on the scatter plot correspond to a specific property, with the X and Y axes representing dimensions derived from factor analysis (linear combinations of variables). The proximity between points signifies similarity in the prevalence of *Trypanosoma* spp. and *Babesia* spp. The observed clusters indicate properties that share similar characteristics. The properties included in this analysis are: Barcelona, Bariri, Base Aerea, Bella Vista, Brisas del cocoa, Campo Hermoso, El Palmar, El Recreo, El Refugio, El Rocio, El Rodeo, Granja El Hachon, La Feria, La Fortuna, La Fortuna IV, La Palma, La Primavera, Las Garzas, Las Margaritas, Marsella, Mi Llanura, Nieves, San Francisco, San Marcos, San Pedro, Santa Ana, Santa Lucía, Universidad de los Llanos and Urania

**Fig. 5:**
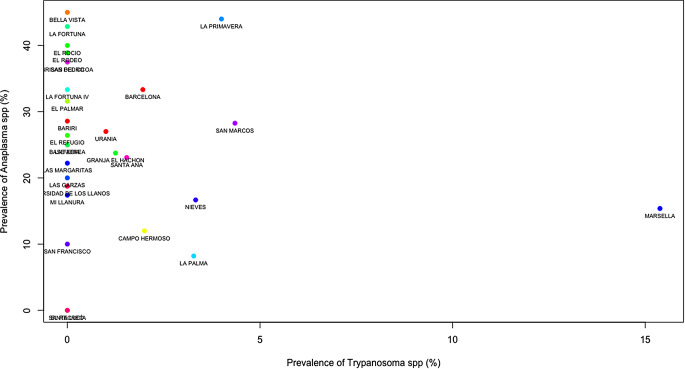
Scatter Plot of properties by the prevalence of *Trypanosoma* and *Anaplasma*: All the points on the scatter plot correspond to a specific property, with the X and Y axes representing dimensions derived from factor analysis (linear combinations of variables). The proximity between points signifies similarity in the prevalence of *Trypanosoma* spp. and *Anaplasma* sp. The observed clusters indicate properties that share similar characteristics. The properties included in this analysis are: Barcelona, Bariri, Base Aerea, Bella Vista, Brisas del cocoa, Campo Hermoso, El Palmar, El Recreo, El Refugio, El Rocio, El Rodeo, Granja El Hachon, La Feria, La Fortuna, La Fortuna IV, La Palma, La Primavera, Las Garzas, Las Margaritas, Marsella, Mi Llanura, Nieves, San Francisco, San Marcos, San Pedro, Santa Ana, Santa Lucía, Universidad de los Llanos and Urania

The dendrogram produced by hierarchical clustering exhibited a strong clustering effect among the properties that tested positive for hemoparasites. Properties with high prevalence were distant from those with lower prevalence and displayed distinct characteristics. Conversely, properties with approximately 10% prevalence of hemoparasites showed a shorter distance between them and shared similar characteristics (Fig. 2). To reinforce this data, a scatter analysis using Kmeans was conducted to demonstrate how the prevalence of hemoparasites varies depending on each property. The findings revealed that clustered properties had co-infections with two hemoparasites; the properties were clustered around *Anaplasma* spp. *– Babesia* spp. (Fig. 3), *Trypanosoma* spp. *– Babesia* spp. (Fig. 4), and *Trypanosoma* spp. *– Anaplasma* spp. (Fig. 5).

### Principal component analysis (PCA)

**Fig. 6:**
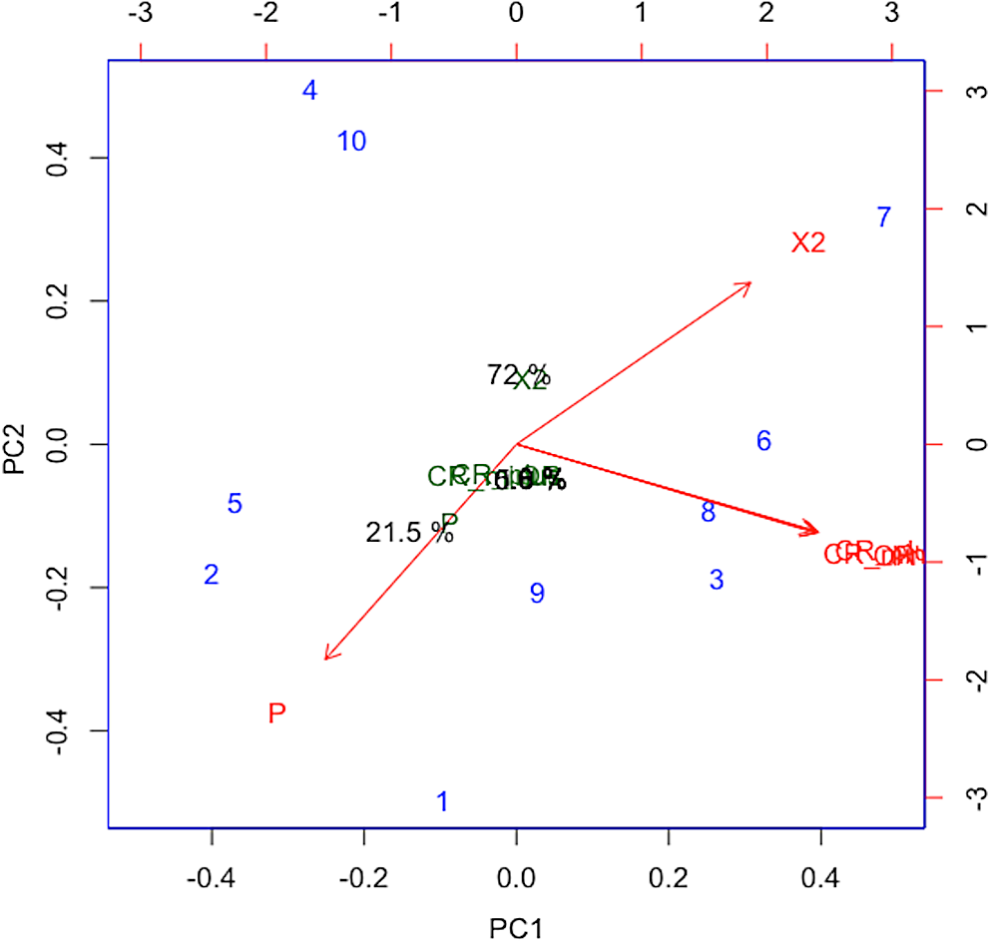
BiPlot of Principal Components Analysis of risk factors: Showing the smaller angles with more positivity and larger angles as negative. The longer arrows indicate more important variables, each blue number represents a variable, in the following order: (1) Cows, (2) Bulls, (3) Jersey breed, (4) Angus breed, (5) Entry of different animal species to the premises, (6) Participates in livestock exhibitions, (7) Burials, (8) use of Palm Kernel and the use of Ivermectin is 9 and Benzimidazole as number 10. The percentage labels on the axes indicate the proportion of total variance explained by each principal component

The PCA developed by Scaled Data evidence different weights of risk factors as significant variables. In the PC1 with high variability, the risk factors such as the presence of cows, use of ivermectin, the use of Jersey of breed and the use of palm kernel show as a principal cluster of risk factors associated with hemoparasitism positivity. The PC 2 related to PC1 show the risk factors such as the use of bulls, the introduction of other animal species to the premises, the use of Angus breed and the use of benzimidazole as co-risk factors that influence the PC1 in the hemoparasites prevalence. Conversely, variables such as burials and participation in livestock exhibitions show a negative correlation between PC1 and PC2 with little influence on the presence of hemoparasitism (Fig. 6**).**

## Discussion

This study identifies for the first time the risk factors associated with infectious cattle diagnosed with *Anaplasma* spp., *Babesia* spp., and *Trypanosome* spp., having a single or co-infection in an endemic area. This identifies the infectious cattle actively involved in the maintenance of endemism because the blood smear technique was able to identify the hemoparasites microscopically at high parasitemic loads. The primo-infected calves under field conditions in Colombia reached the microscopic umbral in the range of 5–10% of erythrocytes parasitized during 3 and 7 weeks after natural infection by *Babesia* spp. and *Anaplasma* spp., respectively (Todorovic and González 1975). Similarly, cattle primo-infected with *Trypanosoma* spp. reached the microscopical detection umbral 3 days after infection, showing the highest sensibility to *T. vivax* under field conditions (Betancourt et al. 1979; Agudelo et al. 1984). These findings confirm that the cattle diagnosed in this study were in the infectious stage because the cattle without this condition have low parasite loads and hemoparasitism must be microscopically undetectable (Böse et al. 1995; Desquesnes et al. 2022).

The overall prevalence of 33.40% corresponds to infectious cattle with high parasite loads and contrasts with previously exposed animals from previous serological surveys in the same area, confirming that these animals represent a small proportion of the herd, leading to the assumption that the rest of the cattle sampled resulted in low parasite loads that were microscopically undetectable and confirming that the small proportion of cattle in endemic areas are infectious (Mahoney and Ross 1972; Regassa et al. 2003). In these areas, the previously exposed animals, undetectable microscopically but detectable by serological methods, represent more than 60% of the animals in the herds, but the infectious animals such as primo-infected, reinfected, or persistently infected animals are easily detected by direct methods and represent a small range of the population ranging between 40% or low in terms of stability or instability endemism (Regassa et al. 2003; Jonsson et al. 2012; Tirosh-Levy et al. 2021). In this sense, the infectious prevalence rate found in this study contrasts with the seroprevalence date of previously exposed animals in the same area, reinforcing the endemic situation according to seroprevalence rates of 69% for *Babesia* spp., 57% for *Anaplasma marginale* (Corrier et al. 1978), and 49% for *Trypanosoma vivax* (Betancourt et al. 1983). However, the infectious cattle in the properties between 16.39 and 53.85% show a relation with seropositive previously exposed cattle of 44.4–100% for *Babesia bovis*, *Babesia bigemin*a, and *Anaplasma marginale* in the same area, suggesting that infectious animals contribute to the spreading of the agent’s inducing endemism that could be stable or unstable related to risk factors in each farm (Mateus and Vizcaíno 1986).

In cases of unstable endemicity, sporadic hemoparasite outbreaks may occur, while in cases of stable endemicity, the enzootic cycle transmission seems to be linked strongly to management, husbandry, and environmental conditions (Alonso et al. 1992). However, husbandry management is associated with vector development and their participation in hemoparasite transmission, this study shows that farms with burials in their lands are risk factors, as well as those including supplements with palm kernel and the use of ivermectin, are all risk factors that contribute to the success of the cycle of biting flies and ticks. The frequent use of ivermectin must be associated with arthropod vector resistance which reinforces the beginning of integral strategies, such as the management of the non-parasite stage of biting flies and the implementation of vaccines for ticks that could be more user-friendly than the use of chemical products (Tafur-Gómez et al. 2020; Villar and Schaeffer 2022). Similarly, the co-circulation of vectors simultaneously is related to the infectious rate of cattle identified by *Anaplasma* spp. at 26.20%, *Babesia* spp. at 8.40%, and *Trypanosoma* spp. *at* 1.30% in 97% of the farms, with main co-infections involving of *Anaplaama* spp. *- Trypanosoma* spp. followed *by Babesia* spp. *- Trypanosoma* spp. verified by cluster analysis. Because the climatic condition in the area favours the biting flies showed higher incidences during the year’s second humid period, especially from August to December (Cassalett et al. 2013). Similarly, with the beginning of the dry season, the weather conditions are favourable for *Rhipicephalus. (Boophilus) microplus* infestation in the transitions of wet to dry and vice versa reaching five peaks of tick along the year and a higher infestation from July to October (Betancourt 1990; Cassalett et al. 2013; Urrea et al. 2019). This vector infestation incidence in cattle relates to the sampling period, and the agents transmitted are related to infectiousness in cattle.

The constant movement of cattle for different porpoises in the involvement area showed a risk factor related to introducing persistently infected cattle on the farms. So, these cattle reach a parasitemic infectious load detectable in a blood smear and act as infection sources favouring agent vectorization and infection reproduction (Antonio Alvarez et al. 2019). Previous studies under artificial infections showed that persistent infected and acute cattle developed a similar parasitemic load in circulation blood, favouring the transovarial transmission of *Babesia* in ticks, with detectable kinetes in the hemolymph of engorged females (Howell et al. 2007). Previously, the ticks of the studied area showed *Babesia* spp. in 2.15% of engorged females, revealing high parasitism for *Babesia bigemina* (Benavides 1987). In other areas of the country, *T. nebulosus* showed participation in transmitting *Trypanosoma vivax* with the identification of this protozoan in the midgut, and *T. occidentalis* showed the *Trypanosoma vivax* in salivary glands (Otte and Abuabara 1991; Parra-Henao et al. 2008). These facts explain the success of hemoparasites transmission and the vectorization involving ticks and biting flies in the studied area.

Regarding the management, sex, and age of infected cattle shows that cows are a risk factor for hemoparasites mainly involving *Anaplasma* spp. and *Babesia* spp., and the presence of cattle older than three years in the farms are a risk factor for *Babesia* spp. These findings agree with previous studies that showed more impact of hemoparasitism in cattle until two years of age, suggesting that cattle in the studied area repeated the arthropod infestation and hemoparasites reinfection until two years, and the cattle older than three years could remain persistent infected for *Babesia* spp. (Jonsson et al. 2012; Giglioti et al. 2018; Antonio Alvarez et al. 2019). Similarly, benzimidazole as a protective factor could be related to cattle coinfected with gastrointestinal parasites, best husbandry management, and the impact of deworming on the immune status of cattle from one or two years old. These facts suggest that immune status is essential to controlling arthropod infestation and hemoparasite progression because multiparasitism and co-infection could immunocompromise cows and calves (Vaumourin et al. 2015). Similarly, the positive cattle share the same grazing areas with buffaloes and canines suggesting that the co-existing of these animal species can intervein as a risk factor due to a related source of infection, previous studies demonstrated the role of buffaloes as a source of infection for *Babesia* spp. and *Trypanosoma* spp. in cattle (Garcia et al. 2016). Crossbreeds and Jersey’s breeds are risk factors related to poor management that maintains persistently infected animals contributing to reproducing infections.

Considering that PC analysis showed in the PC1 risk factors such as the presence of cows, use of ivermectin, the use of Jersey breed and the use of palm kernel as a principal cluster of risk factors associated with hemoparasitism in the cattle and making them relevant to the presence of hemoparasites. PC2 clusters co-risk factors that if implemented in the premises with the PC1 risk factors, could increase in the hemoparasitism prevalence. On another hand burials and participation in livestock exhibitions have a negative correlation between PC1 and PC2, this could be associated to that not most cattle farms implement these variables. This suggests that the development of integrated management of hemoparasitism must consider PC1 and PC2 as principal risk factors and co-factors that contribute to the presence of hemoparasites. Aligned with these results, the implementation of an integrated management strategy considering those risk factors could contribute to the control of hemoparasites. Similarly, previous research in tropical areas such as Ethiopia suggests that the introduction of periodic checkups with regular blood testing to detect infections early in the integrated management strategy contributes to controlling the hemoparasites in cattle (Sitotaw et al. 2014). These findings reinforce the importance of implementing a strategy of control based on the detection of hemoparasites and risk factors.

## Conclusions

We conclude that cattle in the study area found infectiousness and the husbandry management and environment favour the agents’ transmission-inducing enzootic cycle or epizootic cycle with sporadic outbreaks linked to stable or unstable endemism in the herds. Infectious animals diagnosed by parasitological direct methods contribute to checking the endemic degree in the properties. Understanding and addressing co-infections in cattle is of paramount importance for several reasons. First, co-infections can lead to more severe and complicated diseases in cattle. Accurate identification and a fast diagnosis of the pathogens involved are crucial for effective treatment and disease management for economic wellness. These findings reinforce the importance of preventive and integrated strategies to control the vectors and borne pathogens and the importance of introducing technical support for cattle management in tropical areas.

## Data Availability

The authors confirm that the employed data supported the published claims, and the datasets analyzed during the study are available with the corresponding author by reasonable request.
